# What Differs between Patients under Methadone and under Buprenorphine for Opioid Use Disorder (OUD) in Daily Clinical Practice in France? A Short Report

**DOI:** 10.3390/ijerph18041425

**Published:** 2021-02-03

**Authors:** Morgane Guillou-Landreat, Antoine Dany, Gaëlle Challet-Bouju, Edouard Laforgue, Juliette Leboucher, Jean Benoit Hardouin, Caroline Victorri-Vigneau, Marie Grall-Bronnec

**Affiliations:** 1 INSERM UMR 1246, SPHERE, Methods in Patient-Centered Outcomes and Health Research, Nantes and Tours Universities, 44000 Nantes, France; morgane.guillou@chu-brest.fr (M.G.-L.); gaelle.bouju@chu-nantes.fr (G.C.-B.); edouard.laforgue@chu-nantes.fr (E.L.); jean-benoit.hardouin@univ-nantes.fr (J.B.H.); caroline.vigneau@chu-nantes.fr (C.V.-V.); 2 EA 7479 SPURBO, Universite Bretagne Occidentale, 29200 Brest, France; antoine.dany@univ-brest.fr; 3 HUGOPSY Network, 35000 Rennes, France; 4 CHU Nantes, Addictology and Psychiatry Department, Nantes University Hospital, 44000 Nantes, France; juliette.leboucher@chu-nantes.fr; 5 CHU Nantes, Pharmacology Department, Nantes University Hospital, 44000 Nantes, France

**Keywords:** opiate medication treatment, buprenorphine, methadone, daily clinical practice

## Abstract

(1) Background: Opioid use disorder (OUD) is a complex condition that can require long-term treatment. Pharmacological therapy for OUD involves treatment with opioid agonists (OMT) tailored to individual profiles. The aim of our study in daily clinical practice was to compare the profiles of patients treated with methadone (MTD) and those using buprenorphine (BHD or BHD-naloxone-NX). (2) Methods: A cross-sectional multicentre study explored the psychological, somatic and social profiles of patients with Opioid Use Disorder (OUD) following Opioid Maintenance Treatment (BHD, BHD/NX, or MTD). Descriptive and comparative analyses were performed (3) Results: 257 patients were included, a majority were men using heroin. 68% (178) were on MTD, 32% (79) were on BHD. Patients with MTD were significantly more likely to report socio-affective damage, and more likely to be younger and not to report oral or sublingual use as the main route for heroin or non-medical opioids (4) Conclusions: In daily clinical practice, regarding OUD damage, only socio-affective damage was significantly more prevalent among patients on MTD than among those on BHD in the multivariate model. Age and route of administration also differed, and our results could raise the issue of the type of OMT prescribed in case of non-medical use of prescribed opioids. These hypothesis should be confirmed in larger studies.

## 1. Introduction

Opioid use disorder (OUD) is characterized by the repeated use of opioids with harmful consequences [1], and is associated with increased early morbidity and mortality [2]. 

Treatment for OUD combines psychosocial interventions and pharmacotherapy, including Opioid Maintenance Treatment (OMT): Methadone (MTD) and Buprenorphine (BHD) or Buprenorphine-naloxone (BHD/NX). The main goals of OUD treatment focus on harm reduction and improvement of psycho-social and health outcomes [3]. The pharmacological characteristics differ: MTD is a full mu agonist and BHD is a partial mu agonist. They are equally efficacious and have proved to be efficient in terms of public health [3,4,5].

In France, BHD can be prescribed by any physician, while MTD, listed as a narcotic, is less accessible. Its primary prescription is restricted to physicians operating in specialized units or hospitals. After a period of stabilization, follow-up and prescription can be carried out by any physician [3,4,5]. BHD or BHD/NX are considered first–line treatments, accessible for any patients, whereas MTD is identified as a second-line treatment when BHD treatment fails, for patients who inject drugs or for patients with comorbidities [3,6]. 

There is no specific molecule that is effective for all individuals [3], and the appropriate choice of the type of medication is part of the integrated treatment plan, involving indication, patient and healthcare professional agreement, and compatibility with treatment goals and specific needs [7]. In daily practice, the choice of OMT may differ from guidelines. It can be thought that patients following MTD treatment have a more severe profile, combining social and somatic aspects with a higher level of addictive disorder. No studies, even after more than 20 years of OMT prescription in France, have explored the current use of OMT among people with OUD in daily clinical practice. The aim of this study was to compare clinical profiles of patients with OUD according to the type of OMT, in a multicenter study in France, and to analyze, in daily practice, the variables associated with the choice of prescription of MTD or BHD or BHD/NX.

## 2. Materials and Methods

### 2.1. Procedure and Ethics

OPAL (NCT01847729) was an observational, cross-sectional, multi-center study. It combined a clinical evaluation [8] and an ancillary pharmaco-genetic study [9]. OPAL was conducted in accordance with the Good Clinical Practice Guidelines and the Declaration of Helsinki and was approved by the local ethics committee. Written informed consent was collected from all participants.

### 2.2. Participants

Participants were recruited in 8 centers specialized in addictive disorders, including one working in prison, and 2 primary care physicians, belonging to a network supporting care for addictive disorders (Western region, France). The study included patients seeking treatment in these centers, aged 18 years or older, receiving OMT (MTD or BHD or BHD/NX) for at least 6 months for OUD (according to the *DSM-IV*, American Psychiatric Association, 2014). Non-inclusion criteria were difficulties understanding French and legal guardianship. 

### 2.3. Measures

The clinical evaluation consisted of a hetero-assessment (structured interview conducted by one of the investigators) and a self-assessment (self-administered questionnaires completed by the patient). Different clinical data were collected, including screening for impulsiveness and ADHD. The results have been published previously [8]. Only variables that were used for the present analyses are presented in this article:

#### 2.3.1. Sociodemographic Characteristics

We collected data on gender, age, education level, marital and parental status, housing, social support, professional status, and financial situation.

#### 2.3.2. OUD Characteristics 

Data concerning the main substance used was collected using closed, multiple-choice questions on the type of opioid used (heroin, non-medical use of codeine, morphine, buprenorphine, and methadone), age at the initiation of opioid use, age when the opioid dependence appeared, age at the first attempt to quit, the main route of administration (nasal, intravenous, inhaled, oral), strategies used previously to attempt to quit opioid use, and type of damage (negative consequences) related to opioid dependence (financial, socio-affective (familial, relational), psychiatric, professional, legal, physical problems) self-reported by the patients. OMT characteristics were also collected (type of medication, duration). 

#### 2.3.3. Use of other substances and gambling habits 

Patients were asked about any current co-addictions (nicotine/alcohol/gambling/illicit substance use. Current co-addictions were considered in case of nicotine dependence (Fagerström nicotine dependence test [10]), and/or high-risk substance use and/or alcohol misuse (CRAFFT [11]) and/or gambling disorders (The Lie/Bet questionnaire [12]). Detailed methods and results for each co-addiction have been published previously [8]. 

#### 2.3.4. Statistical Analysis

All statistical analyses were carried out on R statistical software (R Core Team). *p*-values were considered significant if they were below 5% for all analyses. A descriptive statistical analysis of the socio-demographic and clinical characteristics was conducted. Continuous variables were described by means and standard deviations if Gaussian and by quantiles otherwise. Categorical variables were presented as numbers and percentages. Univariate followed by multivariate logistic regressions were conducted to determine which variables were associated with the type of OMT used (MTD or BHD-BHD/NX). To identify clinical variables at initiation of the current OMT among patients on MTD and to compare them to those on buprenorphine, the OMT (MTD or BHD-BHD/NX) was used as the binary dependent variable, and variables that had a significant *p*-value in univariate analyses were used as the independent explanatory variables. Model fit was assessed using the Hosmer-Lemeshow test.

## 3. Results

### 3.1. Descriptive Analysis of the Sample 

A total of 257 patients were included: 83.6% (*n* = 215) were included in addictive disorders centers, 12.9% (*n* = 33) in primary care settings and 3.5% (*n* = 9) in an addictive disorder center in prison. Three quarters were men. The majority had an education level under 12 years schooling, and were unemployed. Nearly all participants had stable housing, and one third were in a relationship. The prescribed OMT was MTD for 68% (*n* = 178) of the patients and BHD for 32% (*n* = 79). None was receiving BHD/NX. 

### 3.2. Comparative Analysis 

To compare the patients’ clinical profiles at current OMT initiation (MTD or BHD), the different clinical variables at the time of initiation, were compared. Results of the univariate analysis are presented in Table 1. 

### 3.3. Multivariate Analysis-Logistic Regression 

To explore the clinical profiles of patients according to the OMT prescribed (MTD or BHD-BHD/NX), a logistic regression was performed. The resulting multivariate model is presented in Table 2. The Hosmer-Lemeshow test was used to assess the model fit. The *p*-value associated with our data was 0.225 (χ2 = 10.613, df = 8), indicating that a poor fit was not detected. Compared to patients with BHD-BHD/NX, patients with MTD were significantly more likely to report affective damage, to be younger and not to report the main route of heroin or non-medical opioids as oral or sublingual. They were not more likely to report somatic and professional damage.

## 4. Discussion 

In this large sample of patients following OMT for an OUD, it was shown that in daily practice, clinical profiles differed significantly at initiation of the current OMT between MTD and BHD users. Regarding OUD damage, only socioaffective damage was significantly more prevalent among patients on MTD than among those on BHD in the multivariate model. As MTD is recommended as a second-line treatment when BHDtreatment fails, for patients who inject drugs or for patients with comorbidities [3,6], with most of the time a long history of addictive disorders, the fact that the most severe consequences on social or familial relationships were observed among patients under MTD is consistent. 

Psychiatric damage did not differ between the two groups. This result is surprising, as patients with mental health problems have specific needs, and MTD is particularly recommended for this subgroup [13]. One explanation could be that, as damage was self-reported by the patients, they might not have identified comorbid psychiatric disorders as forms of damage in OUD. Another explanation is that regarding harm reduction, BHD can be readily used for complex situations, because it allows low-threshold care. BHD is thus more frequently prescribed than MTD, and more particularly in France, as primary prescription of MTD is not authorized in primary care. However, 50% of primary care physicians reported in 2009 that they saw at least one patient with an OUD per month [14], and a large majority of OMT in France is delivered in primary care [15]. Numerically, primary care physicians encounter more complex patient profiles with OUD than simple patient profiles, and it is then possible to help a patient presenting dual disorders or comorbidities with a simpler protocol like BHD than with a more complex protocol like MTD.

Differences concerning legal and financial damage were not significant. They may be less systematically explored, or identified as clinically irrelevant in a health-related approach, so that it does not influence the type of OMT prescribed. OMT indications seemed to be adapted in relation to global health damage. The objective of the treatment is to improve the overall state of health, to improve physical health and wellbeing so as to limit social and economic harm to individuals and the community and to allow maintenance in community life [3]. MTD or BHD are both indicated with this in mind. However, MTD is more complicated to prescribe and to manage than BHD.

Finally, age and route of administration also differed significantly. Differences relating to the intravenous route were not significant, despite the fact that MTD is recommended for patients with IV habits [3,6]. However, there was a significant difference for the oral or sublingual routes of administration. One explanation is that patients on BHD were more frequently seeking treatment for a non-medical use of prescribed opioids, including morphine, codeine and BHD for 15.1% of the BHD group. We could hypothesize that there is currently no consensus on how to treat patients dependent on prescribed opioids, and that a pragmatic and realistic approach has developed in daily practice.

Long-acting opioid agonist therapy (i.e., BHD +/− naloxone or methadone), provides an alternative strategy in case of OUD resulting from a non-medical use of a prescribed opioid, as part of comprehensive care with a good therapeutic alliance [3]. In our study, although the choice of the treatment should depend on several determinants [3], BHD seemed to be more frequently prescribed for this indication. The mean age was also older in the BHD group. Indeed, it has been clearly shown that patients with an OUD resulting from prescribed opioids are older than opiate-dependent patients.

### Strengths and Weaknesses

This study collected a large multicenter dataset concerning patients with an OUD and on OMT in daily clinical practice. This provided heterogeneity across the population. Missing data was marginal. Our study does however present limitations. Firstly, its design was cross-sectional, and variables were self-reported by patients. Patients were recruited in centers specialized in addictive disorders or in primary care settings trained for OUD management, but data regarding their addiction treatment histories was not collected. BHD-NX was not included in the analysis, as no patient included had this treatment, but in France, despite its marketing in 2012, BHD-NX still accounts for only 5% of BHD/BHD-NX prescriptions [16]. Secondly, we developed an ad hoc structured interview to explore OUD characteristics (especially the damage incurred). This decision was made because no validated scale was available. 

## 5. Conclusions 

In daily clinical practice, the profiles of patients on MTD or buprenorphine differed significantly. In addition, our results also raise the issue of the non-medical use of prescribed opioids and the indications and type of OMT to implement in this case. In pragmatic daily clinical practice in this study, BHD seemed to be more frequently prescribed in this context. These results should be confirmed in later studies. The choice of the OMT could be motivated by a global health evaluation, by the clinical characteristics of the opioid use disorder, by the patient’s choice, and also by the availability of BHD or MTD, partly as a result of OMT regulations. Changes in clinical practices regarding OMT management in OUD should be taken in account by Public Health authorities to adapt recommendations and to promote new treatment strategies in OUD.

## Figures and Tables

**Table 1:** Univariate comparative analysis of variables at Opioid Maintenance Treatment (OMT) initiation between OMT strategies (methadone (MTD)/Buprenorphine (BHD)).

Variables	MTD (*n* = 178)	BHD (*n* = 79)	*p*
Socio-demographic characteristics			
Gender (%-*n* males)	74.7% (133)	74.7% (59)	1
Current Age (y)	33.8	37.2	<0.001
Age at opioid initiation (y)	20.3	20.9	0.33
Age at opioid dependence (y)	22.3	23.9	0.14
Age at first attempt to quit (y)	25.4	27.3	0.07
Previous strategies for attempting to quit (%-*n*)			0.13
*History of opioid withdrawal*	38.6% (68)	61.3% (39)	
*History of previous OMT prescription*	49.3% (108)	50.6% (40)	
Living conditions (%-*n*)			
Marital status (% living as a couple)	37.6% (67)	42.3% (33)	0.49
Stable housing	88.1% (156)	92.3% (72)	0.38
Social support	92.6% (164)	92.3% (72)	1
Education attainment > 12 y (%)	13.2% (23)	14.4% (11)	0.69
Work status and financial situation % (*n*)			
Active workers	42.7% (76)	52.5% (41)	0.3
Debt	39.7% (70)	31.1% (24)	0.20
Opioid use disorder characteristics			
Opioid mainly used			0.047
*Heroin*	93.2% (165)	84.8% (67)	
*Non-medical codeine or morphine*	3.9% (7)	11.3% (9)	
*Non-medical buprenorphine*	2.8% (5)	3.8% (3)	
Main route of administration of heroin or non-medical opioid			0.028
*Nasal*	69.0% (107)	68.0% (49)	
*Intravenous*	29.0 (45)	22.2 (16)	
*Oral*	1.9% (3)	9.7% (7)	
Type of OUD damage			
*Financial*	75.3% (134)	63.3% (50)	0.05
*Social-affective* *(familial, relationnal)*	77.5% (138)	54.4% (43)	<0.001
*Psychiatric*	72.4% (129)	62.0% (49)	0.10
*Professional*	61.8% (110)	41.7% (33)	0.004
*Legal*	53.3% (95)	40.5% (32)	0.06
*Somatic*	37.6% (67)	21.5% (17)	0.01
Associated problematic substance use			
Alcohol	57.1% (80)	46.7% (29)	0.22
Amphetamine	25.5% (22)	21.7 % (5)	0.79
Barbiturate	29.8% (23)	16.6 (5)	0.22
Cannabis	29.4% (38)	18.8 (10)	0.19
Cocaine	36.9% (44)	24.4 (10)	0.18
LSD	27.5% (22)	15.0% (3)	0.38

OMT: opioid maintenance therapy; OUD: opioid use disorder.

**Table 2:** Model of clinical profiles of patients on MTD compared to those on BHD *n* = 227.

Variable	OR [2.5–97.5%]	*p*-Value
Somatic damage	2.25 [0.96–5.60]	0.069
Socio-affective damage	2.63 [1.35–5.15]	0.004
Professional damage	1.80 [0.96–3.41]	0.069
Age	0.94 [0.90–0.98]	0.004
Main route of administration of heroin or non-medical opioid: oral or sublingual	0.22 [0.04–0.89]	0.04
Main route of administration of heroin or non-medical opioid: IV	0.98 [0.38–2.48]	0.9

## Data Availability

Data are available on reasonable request to the corresponding author.
